# Heat source free water floating carbon nanotube thermoelectric generators

**DOI:** 10.1038/s41598-021-94242-0

**Published:** 2021-07-19

**Authors:** Tomoyuki Chiba, Yuki Amma, Masayuki Takashiri

**Affiliations:** Department of Materials Science, Tokai University, Hiratsuka, Kanagawa 259-1292 Japan

**Keywords:** Thermoelectrics, Carbon nanotubes and fullerenes

## Abstract

Thermoelectric generators (TEGs) produce electric power from environmental heat energy and are expected to play a key role in powering the Internet of things. However, they require a heat source to create a stable and irreversible temperature gradient. Overcoming these restrictions will allow the use of TEGs to proliferate. Therefore, we propose heat source-free water-floating carbon nanotube (CNT) TEGs. Output voltage and power are generated by the temperature gradient in the CNT films in which water pumping via capillary action leads to evaporation-induced cooling in selected areas. Furthermore, the output voltage and power increase when the films are exposed to sunlight and wind flow. These water-floating CNT TEGs demonstrate a pathway for developing wireless monitoring systems for water environments.

## Introduction

Thermoelectric generators (TEGs) produce electricity from heat energy^1–4^, with conventional TEGs connecting n- and p-type materials in series via metal electrodes^5–8^. Placing one end of a TEGs on a heat source induces a temperature gradient in the materials, with heat flowing in the direction along the temperature gradient. Additionally, electrons (n-type materials) and holes (p-type materials) move simultaneously in the same direction of heat flow. Thus, current passes through the TEGs, generating electric power.

TEGs have been proposed as power supplies for wireless sensors and wearable devices that constitute the Internet of things (IoT)^9–12^. These applications require TEGs that are small, lightweight, and mechanically flexible, without excessively high power generated by a large temperature gradient. Thin-film TEGs on a flexible substrate are the most promising candidates to satisfy these requirements^13–16^. Bismuth telluride-based alloys have emerged as a suitable alternative for thin-film TEGs owing to their impressive thermoelectric properties near 300 K^17–20^. Considerable research has been devoted to increasing the thermoelectric performance and optimizing the structure of TEGs^21–24^. However, current TEGs suffer from two major weaknesses. First, a heat source is required to create the temperature gradient in the TEGs. Second, heat flows vertically to the heat source and the direction cannot be controlled without proper placement of the heat source and heat sink; therefore, p- and n-type materials are required to produce electric power efficiently. Overcoming these weaknesses will help increase the applications of TEGs.

**Figure 1:**
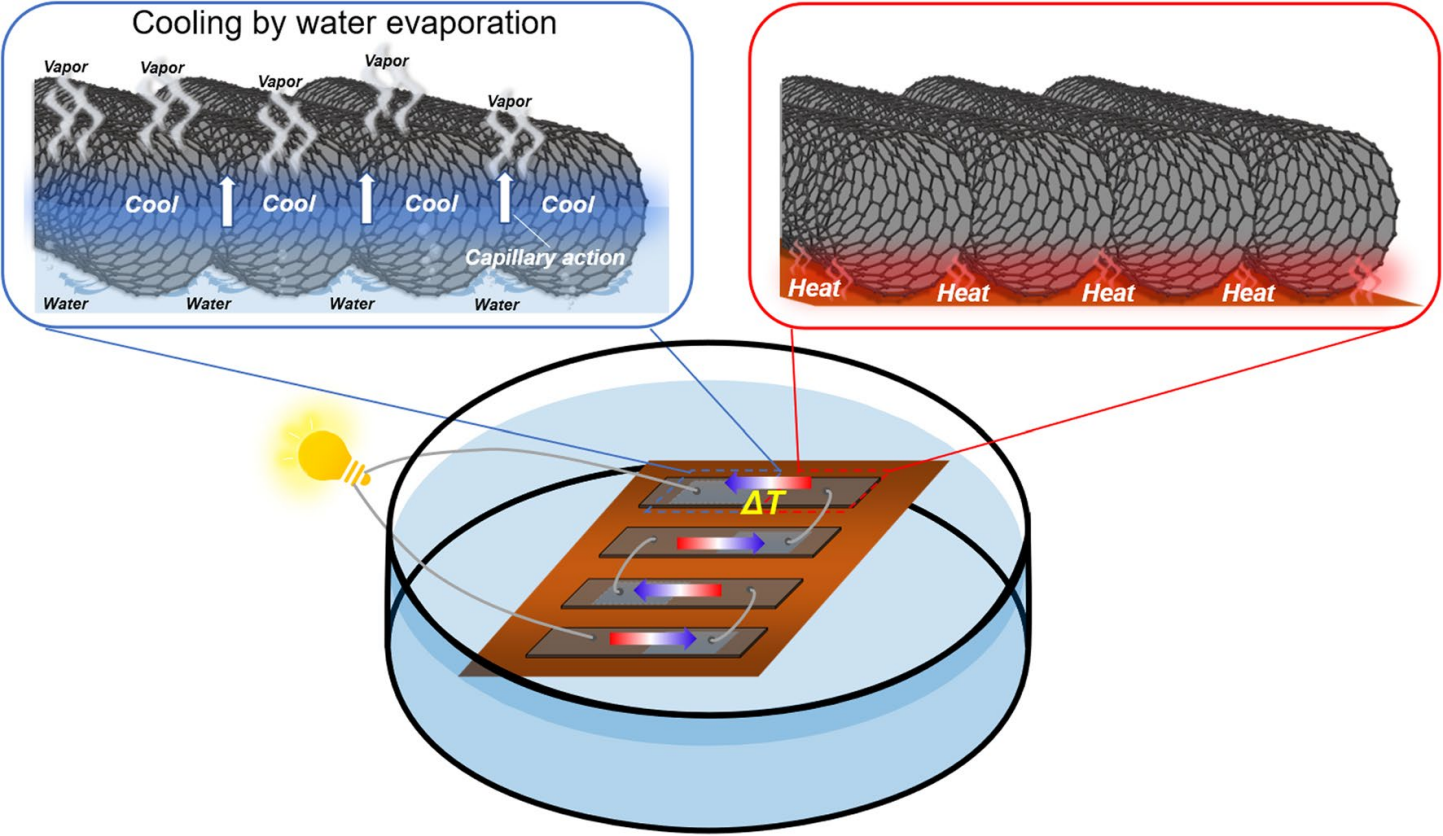
Design principle of water-floating SWCNT film TEGs. Each thin film is divided into water-permeable and water-nonpermeable areas. In the former, water passes through the gaps in the SWCNT bundle to reach the surface via capillary action. The surface temperature is reduced via an evaporation-induced cooling. Thus, a temperature gradient is created in each film, with output voltage generated via the Seebeck effect.

One promising TEG structure for surmounting these obstacles is water-floating carbon nanotube (CNT) TEGs (see Fig. 1). Carbon nanotubes are lightweight and possess both flexibility and mechanical strength^25–27^. In addition, single-wall carbon nanotubes (SWCNTs) with specific chirality exhibit relatively high thermoelectric properties near room temperature^28–32^. When a bundle of SWCNT films floats on water, the water passes through the gaps in the SWCNT bundle and reaches the surface via capillary action. When the water evaporates, heat is absorbed, and the surface temperature drops via evaporation-induced cooling. Moreover, this cooling effect can be enhanced by exposing the SWCNT films to sunlight and wind flow. Designing the SWCNT films to have permeable and nonpermeable areas establishes a temperature gradient between these areas, enabling the generation of output voltage via the Seebeck effect. This phenomenon suggests that TEGs can be fabricated relatively easily using either p- or n-type materials alone. Typically, SWCNTs exhibit stable p-type properties^33^. In contrast, synthesizing air-stable n-type SWCNTs is highly challenging^34–37^. Therefore, water-floating TEGs based on p-type SWCNT films are promising candidates to supply power to wireless environmental sensors used to measure the quality, quantity, and temperature of water. In addition, the TEGs can be potential used as wearable sensors where body sweat represents the water source.

Here, we report the fabrication and successful testing of water-floating TEGs using only p-type SWCNT films. The flexible substrate comprises square holes arranged in a staggered pattern and each hole is covered by an edge of a rectangular SWCNT film. The ends of adjacent SWCNT films are connected by metal electrodes. The temperature distribution in the TEGs was monitored as they floated on water. Thus, we verified that the heat flow can be controlled by evaporation-induced cooling and that the temperature gradient is stable. In addition, the output voltage and power generated by the TEGs was measured in response to variations in water temperature, sunlight exposure, and wind exposure.

### Fabrication and testing of water-floating SWCNT film TEGs

**Figure 2:**
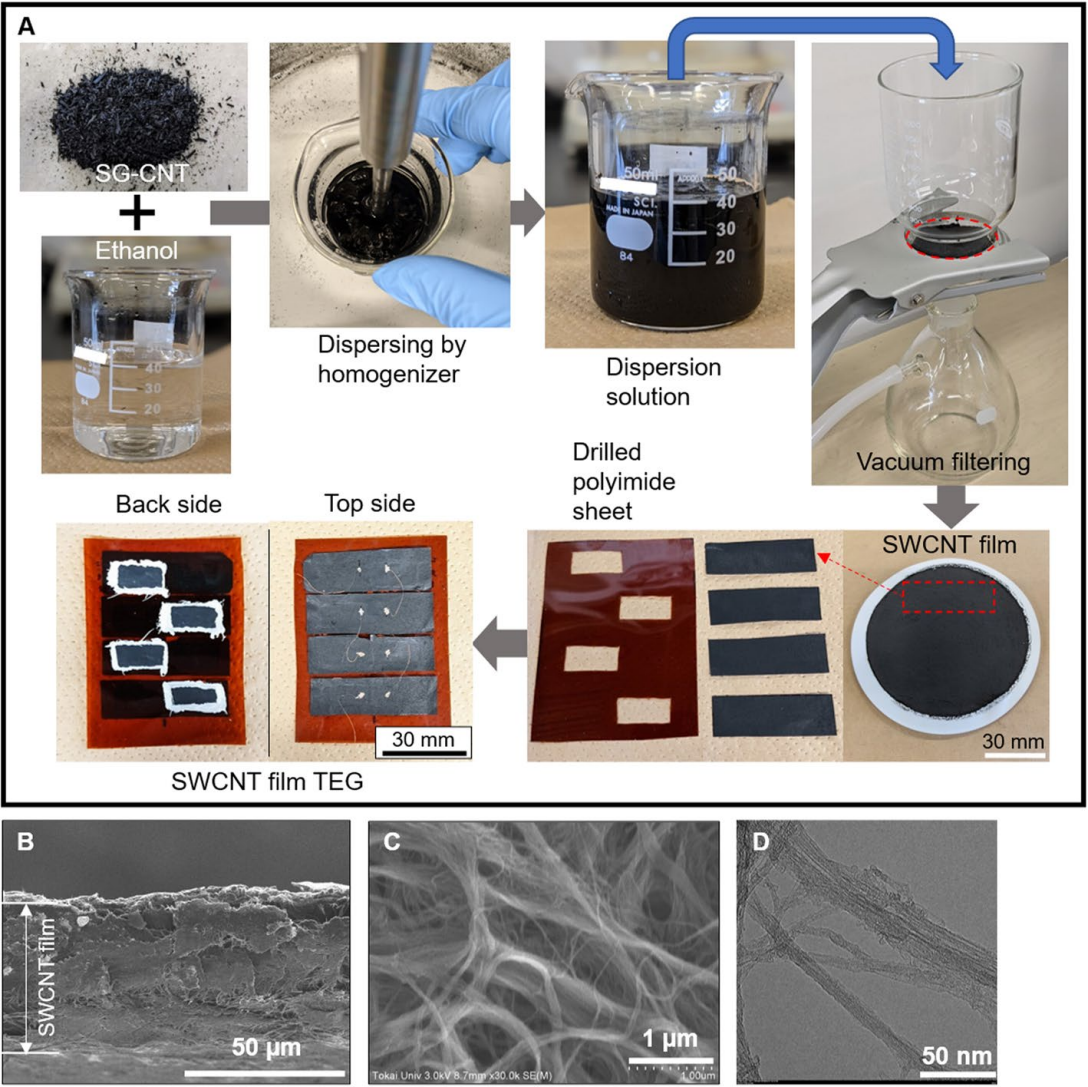
Fabrication and structural characterization of the water-floating SWCNT film TEGs. (**A**) Photographs showing the synthesis of SWCNT film TEGs. (**B**) SEM cross section of an SWCNT film. (**C**) SEM image of an SWCNT film surface. (**D**) TEM image of SWCNTs.

The process used to fabricate the water-floating SWCNT film TEGs is illustrated in Fig. 2A. Carbon nanotubes synthesized by the super-growth method (SG-CNTs) (ZEONANO SG101, ZEON Co.) were used as the starting material^38,39^. Powdered SG-CNTs were dispersed in ethanol to prepare an SG-CNT dispersion solution with a concentration of 0.2 wt%. Next, an ultrasonic homogenizer (SONICS 85, AZONE Co.) was used to disperse the SG-CNT powder completely. The output power of the homogenizer was 60 W, and the dispersion time was 20 min. Because the vibrational energy during dispersion generates heat, the dispersion was conducted in a cold-water bath. The SWCNT films were prepared by a vacuum filtering method. A membrane filter (PTFE, 90 mm diameter: ADVANTEC) was placed in a filter holder in a suction bottle, and the dispersion solution was filtered by reducing the pressure in the suction bottle using a rotary pump to extract the material in the solution. A CNT-dispersed solution (40 mL) was released drop-by-drop onto the filter and aspirated for 1 h to produce SWCNT films with a diameter of approximately 80 mm. After drying for 24 h in air, the SWCNT films were removed from the membrane filter. To assemble the TEGs, the SWCNT films were cut into four pieces, each measuring 45 mm in length and 15 mm in width. The substrate (80 mm × 60 mm, 125 µm thick) was a polyimide sheet (Kapton, DuPont) in which four rectangular holes (20 mm × 10 mm) were drilled in a staggered arrangement. The four sections of the SWCNT films were bonded to the substrate with silver paste such that adjacent films each were half covered by a hole in the polyimide. The SWCNT films were connected in series using thin copper wires. The total resistance of the TEGs was 18.5 Ω, of which the resistance of the Cu wire alone was 0.5 Ω. Therefore, the power loss due to the resistance of the Cu wires was estimated to be approximately 2.7%.

The cross-plane SEM image shows that the film was deposited almost uniformly, with a film thickness of approximately 50 µm (Fig. 2B). The surface SEM image shows the stacking of numerous winding SWCNT bundles with various diameters, including the presence of gaps between the bundles (Fig. 2C). These gaps allow water to reach the surface via capillary action. The TEM image of the SWCNTs shows that their diameters measure several tens of nanometers, with the SWCNTs arranged in a one-dimensional linear structure (Fig. 2D).

We conducted the experiments assuming typical natural environment conditions. The SWCNT film TEGs were floated on a 450 mL volume of water at initial temperatures of approximately 20 °C and 80 °C. Wind was applied to the TEGs using a compact circulator (PCF-HD15-W, IRIS OHYAMA Inc.) while the wind speed (3.0 m/s) was measured by an anemometer (SP-82AT, Mother Tool Co.). The TEGs were irradiated using an artificial solar illuminator (XC-100, SERIC Ltd.) to simulate direct sunlight (approximate light intensity: 1000 W/m^2^), with the intensity measured by a solar power meter (DT-1307, CEM Instruments). The temperature distribution in the TEGs was measured by a thermographic camera (Type F30W, Japan Avionics). The output voltage was measured using a heat flow logger (LR8432, Hioki Co.).

### Thermoelectric properties of SWCNTs and their films

The thermoelectric properties of the SWCNT films are summarized in Table 1. The in-plane electrical conductivity, Seebeck coefficient, and power factor near 20 °C are 88 S/cm, 55 µV/K, and 26.7 µW/(m K^2^), respectively. Notably, these thermoelectric properties were exhibited by the SWCNT films using the ethanol-based dispersion solution, as mentioned in the last section. For comparison, we prepared the SWCNT films using the water-based dispersion solution containing an anionic surfactant of sodium dodecylbenzenesulfonate. The thermoelectric properties of the SWCNT films using the water-based dispersion solution were an electrical conductivity of 72 S/cm, a Seebeck coefficient of 50 µV/K, and a power factor of 18.7 µW/(m K^2^). The SWCNT films using the ethanol-based dispersion exhibited higher thermoelectric properties than the SWCNT films using the water-based dispersion solution. This is a reason why we used the SWCNT films using the ethanol-based dispersion.

**Table 1:** Thermoelectric properties of SWCNT films.

SWCNT film	*σ* (S/cm)	*S* (μV/K)	*PF* (μW/(m K^2^))	*D* (mm^2^/s)	*κ* (W/(m K))
In-plane	88	55	26.7	18.1	5.4
Cross-plane	–	–	–	0.3	9.0 × 10^–2^

The in- and cross-plane thermal diffusivities, *D*_*in*_* and D*_*cross*_, of the SWCNT films using the ethanol-based dispersion solution are 18.1 and 0.3 mm^2^/s, respectively. The thermal conductivity can be determined from the thermal diffusivity (*D*), density (*ρ*), and specific heat (*C*_*p*_) based on the equation *κ* = *DρC*_*p*_^40^. The density of the SWCNT film was measured as 0.31 g/cm^3^, while a specific heat of 0.96 J/(g K) was considered based on existing literature^41^. The in- and cross-plane thermal conductivities were determined to be 5.4 and 9.0 × 10^–2^ W/(m K), respectively, which are lower than those of SWCNT films reported previously, owing to differences in the electrical conductivity due to the different synthesis methods^42^. However, the lower thermal conductivities of the SWCNT films in this study facilitate effective temperature gradient generation in the films.

### Performance of water-floating SWCNT film TEGs

**Figure 3:**
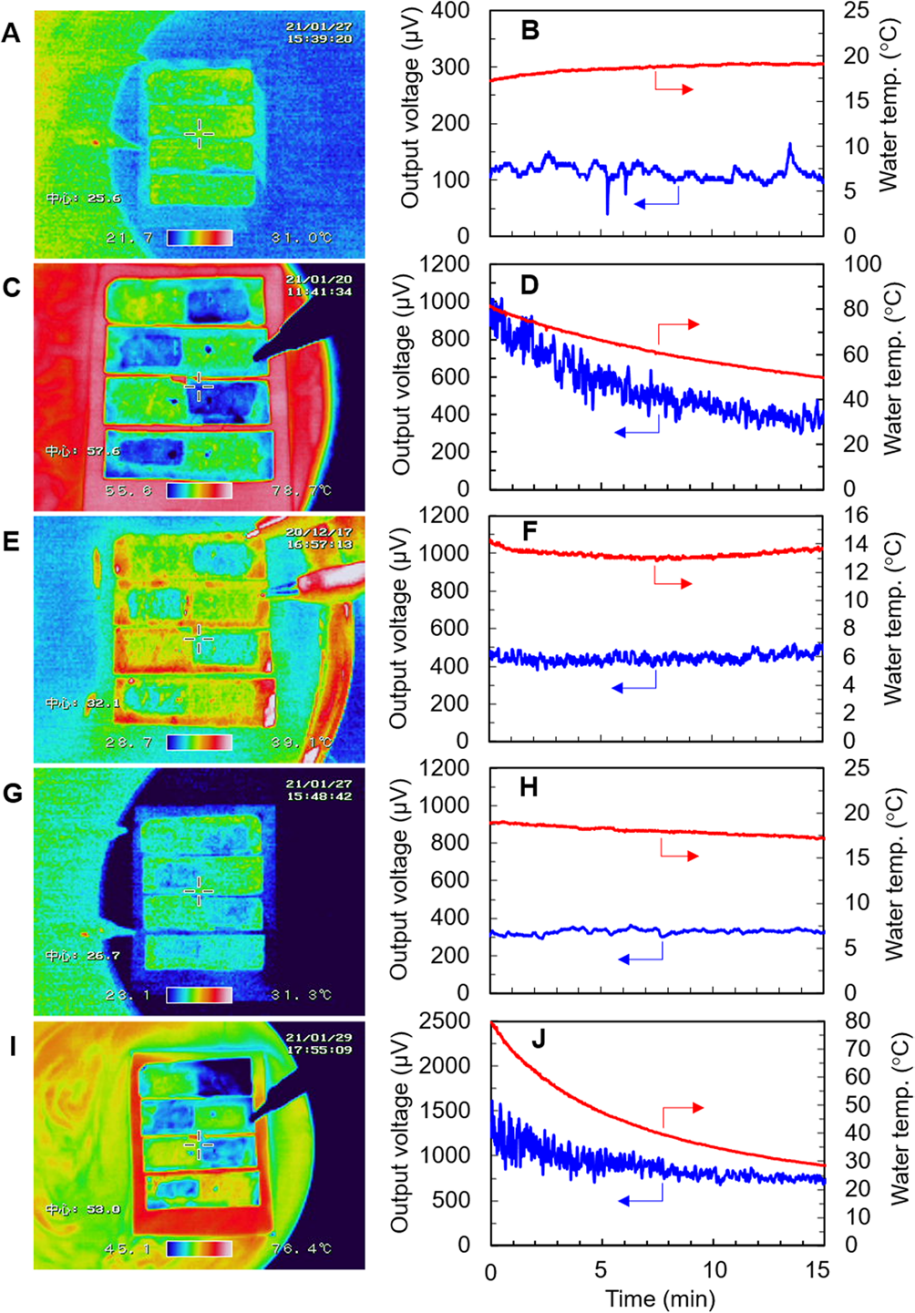
Temperature distribution and performance of the water-floating SWCNT film TEGs for various environmental conditions. Thermographic images of the TEG corresponding to (**A**) no sunlight or wind exposure at an initial temperature of approximately 20 °C, (**C**) no sunlight or wind exposure at an initial temperature of approximately 80 °C, (**E**) simulated sunlight but no wind, (**G**) wind but no simulated sunlight, and (**I**) both simulated sunlight and wind exposure at an initial temperature of approximately 80 °C. (**B**,**D**,**F**,**H**,**J**) Evolution of the output voltage (blue data) and water temperature (red data) corresponding to the conditions in (**A**,**C**,**E**,**G**,**I**), respectively.

The temperature distribution and performance of the water-floating SWCNT film TEGs in response to various environmental conditions are shown in Fig. 3. The ambient temperature in all experiments was approximately 20 °C. The experimental setup is shown in Fig. S1 (Supplementary Material). We confirmed the repeatability of the thermoelectric performances of the devices by repeatably measuring them under the same conditions. For a water temperature of approximately 20 °C, and in the absence of simulated sunlight and wind, the temperature gradients between the positions with and without substrate holes were invisible in the thermographic image (Fig. 3A). However, a stable output voltage of approximately 120 µV was detected with four SWCNT films (Fig. 3B), indicating that an approximate temperature difference of 0.5 K occurred in each film based on the Seebeck coefficient of the SWCNT films (55 µV/K). The output power, *P*, was calculated by the following equation: *P* = *V*^2^/4*R*, where *V* and *R* are the output voltage and resistance of TEGs, respectively. As a result, the approximate output power of 0.2 nW was generated in the TEGs. As shown in Fig. S2 (see Supplementary Material), we verified that an almost constant value of output voltage was maintained when the TEGs floated on the water for 60 min, thereby demonstrating stable output voltage without a heat source. The thermographic image in Fig. 3C shows that for an initial water temperature of 80 °C (without simulated sunlight and wind), a clear temperature gradient is established between the positions with and without substrate holes. Furthermore, the ‘hot’ and ‘cold’ areas are located opposite each other on the adjacent films. This implies that the heat flow can be controlled by changing the position of holes in the substrate and that only one type of (n-type or p-type) film is required to create the TEGs. The approximate temperature difference under these conditions was estimated to be 5 K, and the temperature gradient at edge of the holes is steep because the in-plane thermal conductivity of the SWCNT films (*κ*_*in*_ = 5.4 W/(m K)) is not substantially high. In addition, owing to the low thermal conductivity of the polyimide substrate (*κ* = 0.16 W/(m K)), heat is not transferred between adjacent films. These results demonstrate that the size of the films and the interval between them can be reduced to increase the density of output voltage and power in the TEGs as provided in Fig. S3 (Supplementary Material). The output voltage at a water temperature of 80 °C was 950 µV, and the output power was estimated to be 12.2 nW. The output voltage was observed to decrease with temperature; at a water temperature of 50 °C, the voltage was 400 µV and the estimating output power was 2.2 nW (Fig. 3D). A temperature gradient was also observed when exposing the TEGs to simulated sunlight (1000 W/m^2^) in a wind-free environment at a water temperature of 14 °C (Fig. 3E). A stable output voltage of approximately 450 µV was detected with an output power of 2.7 nW, indicating a temperature difference of 2 K in each film (Fig. 3F), which represented a 3.7-fold increase in the output voltage compared with the corresponding measurement without simulated sunlight and wind. This is attributed to the temperature of the SWCNT films at hole-free locations, which increased because the film exhibited extremely high light absorption, while the temperature of the films at the holes did not increase because of the cooling effect induced by water evaporation. As shown in Fig. S4 (Supplementary Material), we verified that the humidity above the films at the holes was higher than that above the films at hole-free locations. The thermographic image in Fig. 3G, which corresponds to wind flow (3.0 m/s) without simulated sunlight at a water temperature of 18 °C, shows a slight temperature gradient. In addition, a temperature difference between the SWCNT films and the water was also clearly observed. This is because the heat transfer from the water to the SWCNT films was limited by the relatively low cross-plane thermal conductivity of the SWCNT films (*κ* = 9.0 × 10^–2^ W/(m K)). Based on the stable output voltage of approximately 300 µV that was detected under these conditions while the output power was 1.2 nW, we can infer a temperature difference of 1.4 K in each film (Fig. 3H). Exposing the TEGs to a wind flow of 3 m/s increased the output voltage by a factor of 2.5 relative to the wind and simulated sunlight-free measurement. Wind causes the concentration of water vapor near the film surface to decrease. Thus, water evaporation is promoted, which lowers the surface temperature. This suggests that the TEGs can generate the output voltage at night. Our experiments demonstrate that water temperature, sunlight, and wind all affect the generation of output voltage in the TEGs. Therefore, we determined the combination of water temperature, sunlight exposure, and wind exposure required to optimize the generation of output voltage in the TEGs (Fig. 3G). Consequently, we realized an output voltage of 1300 µV and output power of 22.8 nW at a water temperature of 80 °C, simulated sunlight of 1000 W/m^2^, and wind speed of 3.0 m/s. Decreasing the water temperature while maintaining the other conditions resulted in a decrease in the output voltage, with an output voltage of 800 µV and output power of 8.2 nW recorded at a water temperature of 30 °C (Fig. 3H). Finally, we verified that the TEGs could generate the output voltage in actual environmental conditions (natural sunlight and wind) as shown in Fig. S5 (see Supplementary Material).

## Conclusion

We fabricated heat source-free water-floating SWCNT film TEGs. The output voltage and power were generated by the temperature gradient in the SWCNT films, in which water pumping via capillary action leads to evaporation-induced cooling in selected areas. The output voltage and power increased when the films were exposed to sunlight and wind flow. These results demonstrated that improvements could be made by applying various environmental conditions. Furthermore, the output voltage and power could be increased by optimizing the size of the SWCNT films and the holes in the substrate, as well as by increasing the number of smaller films. Therefore, this study provides a vital platform for further investigations to develop heat source-free power supplies for wireless monitoring systems, such as water quality control. In addition, the TEGs can be used as wearable sensors that generate output voltage using the evaporation of sweat from the skin surface. Once this system is realized, the health management and living environment post-COVID‑19 can be considerably improved.

## Methods

The structural properties of the SWCNT films were analyzed using scanning electron microscopy (SEM: S-4800, Hitachi) and transmission electron microscopy (TEM: H7700, Hitachi). The in-plane electrical conductivity, *σ*, of the SWCNT films was measured to 20 °C using a four-point probe method (Napson, RT-70 V). The in-plane Seebeck coefficient, *S*, was also measured to 20 °C using a custom-built instrument^43^. One end of a thin film was connected to a heat sink and the other to a heater. The Seebeck coefficient was determined as the ratio of the potential difference across the membrane to the temperature difference measured using two 0.1 mm diameter K-type thermocouples pressed against the membrane. Then, the in-plane power factor, *σS*^2^, was obtained from the measured electrical conductivity and Seebeck coefficient. The in-plane and cross-plane thermal diffusivities, *D*_*in*_ and *D*_*cross*_, were measured using non-contact laser spot periodic heating radiation thermometry (TA33 thermowave analyzer, Bethel Co.).

## Supplementary Information

Supplementary Information.

## Data Availability

All data are available in the main text or the supplementary materials.
