# Supplementary material for: Heat source free water floating carbon nanotube thermoelectric generators
Source: Sci Rep. 2021 Jul 19;11:14707. doi: 10.1038/s41598-021-94242-0 (PMC8289987; doi:10.1038/s41598-021-94242-0)
Supplement: Supplementary file 1 — Supplementary Information. [file 41598_2021_94242_MOESM1_ESM.docx]

Heat source free water floating carbon nanotube thermoelectric generators

Tomoyuki Chiba^1^, Yuki Amma^1^, Masayuki Takashiri ^1,*^

^1^ Department of Materials Science, Tokai University, Hiratsuka, Kanagawa 259-1292, Japan

* Corresponding author. Email: takashiri@tokai-u.jp (M.T.)

**Supplementary Materials**

**Fig. S1. A photograph of the experimental setup for measuring thermoelectric power.** The SWCNT film TEGs were floated on a 450 mL volume of water at initial temperatures of approximately 20°C and 80°C. Wind was applied to the TEGs using a compact circulator at the wind speed of 3.0 m/s. The TEGs were irradiated using an artificial solar illuminator to simulate direct sunlight at an approximate light intensity of 1000 W/m^2^. The temperature distribution in the TEGs was measured by a thermographic camera. The thermoelectric power was measured using a heat flow logger. The SWCNT films were connected in series using thin copper wires which were fixed with the pressed indium grains.

**Fig. S2. Temperature distribution and performance of the water-floating SWCNT film TEGs for extended conditions.** Thermographic images of the TEG corresponding to (A) no sunlight or wind exposure at an initial temperature of approximately 20°C, (C) both simulated sunlight and wind exposure at an initial temperature of approximately 20°C. (B) and (D) Evolution of the output voltage (blue data) and water temperature (red data) corresponding to the conditions in (A) and (C), respectively.

**
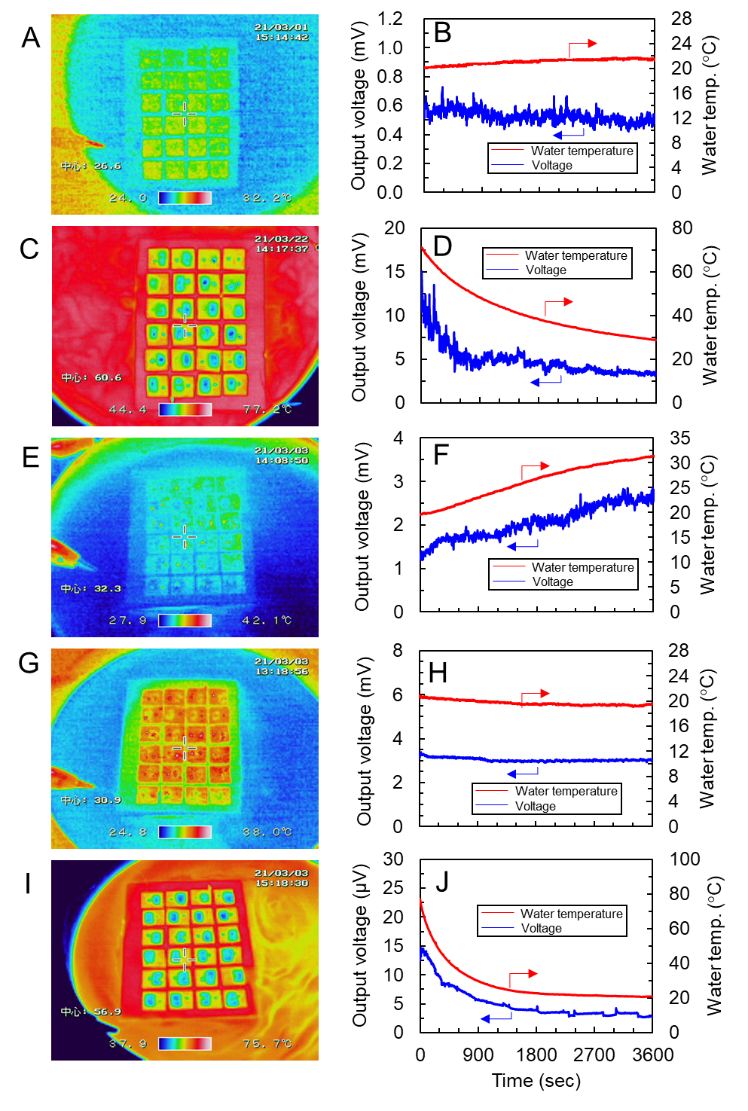
Fig. S3. Temperature distribution and performance of SWCNT film TEGs with 24 sections of SWCNT films.** Thermographic images of the TEG corresponding to (A) no sunlight or wind exposure at an initial temperature of approximately 20°C, (C) no sunlight or wind exposure at an initial temperature of approximately 80°C, (E) simulated sunlight but no wind, (G) wind but no simulated sunlight, and (I) both simulated sunlight and wind exposure at an initial temperature of approximately 80°C. (B), (D), (F), (H), and (J) Evolution of the output voltage (blue data) and water temperature (red data) corresponding to the conditions in (A), (C), (E), (G), and (I), respectively. The size of the polyimide substrate (80 mm × 60 mm, 125 µm thickness) was not changed from its presented in the main article, but the hole size in the polyimide substrate (4 mm × 5 mm) and the SWCNT film size (10 mm × 10 mm) were changed. All the measurement conditions were the same as those performed in the main article, and the ambient temperature was approximately 20°C. The performance of the TEGs was improved compared to that in the main article under all measurement conditions due to the increase in the number of films. We verified that temperature differences were observed in each film even though the holes in the substrate and the SWCNT films were made smaller.

**Fig. S4. Humidity measurement in the SWCNT film TEG at the positions with and without holes in the substrate.** The humidity was measured for 3600 s. The humidity of the area with holes in the substrate was measured from 0 to 1800 s, and that of the area without holes in the substrate was measured from 1800 to 3600 s. (A) shows the humidity of SWCNT film TEG floating in water (W.T. 20°C). (B) shows the humidity of SWCNT film TEG floating in water under artificial solar light (1000 W/m^2^) and wind (3.0 m/s). The humidity measurements in (A) and (B) show that the humidity in the hole area (0 ~ 1800 s) is generally higher than the humidity in the area without holes in the substrate (1800 ~ 3600 s). The overall humidity in (B) is lower than that in (A). This phenomenon occurred because the air flow near the surface of SWCNT film was created by blowing the wind, and the evaporated vapor was ventilated, thus lowering the humidity. The humidity measurement in (B) corresponds to the temperature distribution and performance in the main article.

**Fig. S5. Temperature distribution and performance of the water-floating SWCNT film TEGs for outdoor measurement.** In the outdoor measurements, the effects of natural sunlight variations and wind fluctuations on the temperature distribution and performance of SWCNT film TEG were examined. The temperature distribution in (A)-(C) and the performance in (D) were measured on March 25, 2021 at Hiratsuka city (northern latitude of 35 degrees), Japan. The weather on the day was cloudy (0 min of sunlight during the measurement time), the temperature was 18°C, and the wind speed was 0 ~ 3.1 m/s. The temperature distribution of the SWCNT film TEG was measured every two hours from 11:40 AM to 15:40 PM. In (A) ~ (C), no temperature difference was observed, but the thermoelectric power of 0.3 ~ 0.6 mV was obtained.
